# Pharmacokinetic/pharmacodynamic integration and modelling of oxytetracycline for the porcine pneumonia pathogens *Actinobacillus pleuropneumoniae* and *Pasteurella multocida*

**DOI:** 10.1111/jvp.12385

**Published:** 2017-01-16

**Authors:** L. Dorey, L. Pelligand, Z. Cheng, P. Lees

**Affiliations:** ^1^ Department of Comparative Biological Sciences The Royal Veterinary College Hatfield UK

## Abstract

Pharmacokinetic–pharmacodynamic (PK/PD) integration and modelling were used to predict dosage schedules of oxytetracycline for two pig pneumonia pathogens, *Actinobacillus pleuropneumoniae* and *Pasteurella multocida*. Minimum inhibitory concentration (*MIC*) and mutant prevention concentration (MPC) were determined in broth and porcine serum. PK/PD integration established ratios of average concentration over 48 h (*C*_av0–48 h_)/*MIC* of 5.87 and 0.27 *μ*g/mL (*P. multocida*) and 0.70 and 0.85 *μ*g/mL (*A. pleuropneumoniae*) for broth and serum *MIC*s, respectively. PK/PD modelling of *in vitro* time–kill curves established broth and serum breakpoint values for area under curve (*AUC*_0–24 h_)/*MIC* for three levels of inhibition of growth, bacteriostasis and 3 and 4 log_10_ reductions in bacterial count. Doses were then predicted for each pathogen, based on Monte Carlo simulations, for: (i) bacteriostatic and bactericidal levels of kill; (ii) 50% and 90% target attainment rates (TAR); and (iii) single dosing and daily dosing at steady‐state. For 90% TAR, predicted daily doses at steady‐state for bactericidal actions were 1123 mg/kg (*P. multocida*) and 43 mg/kg (*A. pleuropneumoniae*) based on serum *MIC*s. Lower TARs were predicted from broth *MIC* data; corresponding dose estimates were 95 mg/kg (*P. multocida*) and 34 mg/kg (*A. pleuropneumoniae*).

## Introduction

The tetracycline group of antimicrobial drugs, discovered in 1948, has consistently had the highest veterinary sales volume of the seven drug classes analysed by UK‐VARSS (2014). Resistance to oxytetracycline may be similar to that reported for tetracycline, but this remains to be confirmed. Nevertheless, according to the Clinical Laboratory Standards Institute (CLSI, 2013), whilst tetracycline is the class representative, *MIC* and breakpoint interpretation for tetracycline also apply to oxytetracycline.

Oxytetracycline is a broad‐spectrum drug, classified as a bacteriostat, when administered at clinical dosages, but *in vitro* studies have demonstrated bactericidal actions (Brentnall *et al*., 2012; Dorey *et al*., 2016; Lees *et al*., 2016). Several pathogens implicated in pig pneumonia, including mycoplasma species, were shown historically to be susceptible to the actions of oxytetracycline. It has been registered for veterinary use in most European countries and has been in extensive use in farm animals for more than 60 years. It is therefore still a drug of considerable scientific and clinical interest.

For the treatment of porcine pneumonia, oxytetracycline has the advantage of low cost, clinical efficacy in at least some cases and availability in a long‐acting formulation (Lees & Toutain, 2011). Licensed 20–30% w/v strength parenteral formulations of oxytetracycline have persistent actions, because of the high strength and high dosage used (20–30 mg/kg), leading to sustained absorption from the reservoir site of intramuscular injection. This gives rise to flip‐flop pharmacokinetics (PK) (Nouws & Vree, 1983; Toutain & Raynaud, 1983; Nouws *et al*., 1990). In recent years, there have been major advances in designing dosage schedules of antimicrobial drugs, based on integration and modelling of pharmacodynamic (PD) and PK data. These approaches have provided novel strategies for predicting drug dosages that optimize efficacy and minimize opportunities for the emergence of resistance (Nielsen *et al*., 2011; Martinez *et al*., 2012; Nielsen & Friberg, 2013; Papich, 2014; Rey *et al*., 2014; Vilalta *et al*., 2014; Lees *et al*., 2015b).

The most commonly used (PD parameter to determine potency of antimicrobial drugs is minimum inhibitory concentration (*MIC*); this is the lowest concentration (based on twofold dilutions) that inhibits visible bacterial growth after 16‐ to 24‐h incubation under standard conditions. CLSI guidelines recommend that *MIC* results be established for 16–20 h for most micro‐organisms, except fastidious micro‐organisms such as *Actinobacillus pleuropneumoniae* that require 20‐ to 24‐h incubation (CLSI VET01‐A4 and VET01‐S, CLSI, 2013). Also of increasing interest is mutant prevention concentration (MPC); this is the concentration preventing the growth of the least susceptible cells in high‐density bacterial populations (Blondeau *et al*., 2004). Integration of *in vitro* generated potency indices with *in vivo*‐generated PK data has been used extensively to generate three PK/PD indices, namely the ratios, maximum plasma concentration (*C*_max_)/*MIC* and area under plasma concentration–time curve (*AUC*_24 h_)/*MIC*, and time (*T*) > *MIC*, the time for which concentration exceeds *MIC*.

Integrated PK/PD data are commonly supplemented by *in vitro* time–kill studies. These generate information on the time course of antimicrobial action, enabling classification of drugs as time‐, concentration‐ or co‐dependent in their killing actions. From time–kill studies, numerical values of PK/PD breakpoints can be determined by PK/PD modelling (Aliabadi & Lees, 2002; Toutain & Lees, 2004; Mouton *et al*., 2011; Martinez *et al*., 2012; Nielsen & Friberg, 2013; Papich, 2014; Sidhu *et al*., 2014; Lees *et al*., 2015b). These PD and PK data may be used additionally, with wild‐type *MIC* distributions of susceptible pathogens, to conduct Monte Carlo simulations (MCS) to predict doses providing a range of predetermined levels of kill. Ideally, predicted doses are then correlated with clinical and bacteriological cures in animal disease models and clinical trials (Nielsen *et al*., 2011; Nielsen & Friberg, 2013; Papich, 2014; Lees *et al*., 2015b).

**Figure 1:**
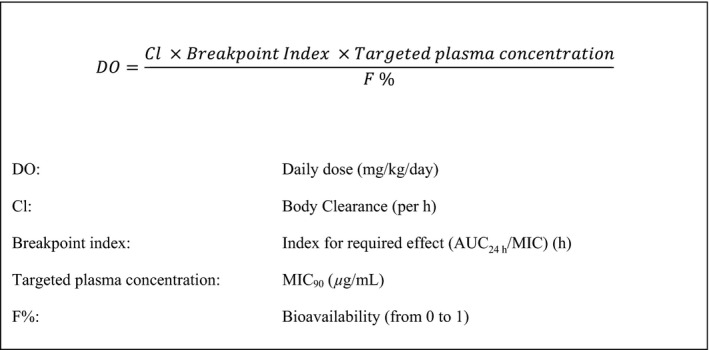
Formula for calculation of daily drug dose based on pharmacokinetic and pharmacodynamic variables.

With the aims of (i) extending the useful life of older antimicrobial drugs and (ii) reducing the likelihood of resistance emergence and ensuring prudent use of these drugs, there have been proposals to re‐evaluate dose schedules that were set, in many instances, many years ago (Aliabadi & Lees, 2001; Mouton *et al*., 2011, 2012; Nielsen *et al*., 2011; Martinez *et al*., 2012; Nielsen & Friberg, 2013; Nyberg *et al*., 2014; Papich, 2014; Rey *et al*., 2014; Toutain *et al*., 2017). The European Union Committee on Antimicrobial Testing (EUCAST) approach to dosage re‐evaluation has been described by Mouton *et al*. (2011). Moreover, the above‐named authors have recognized that a sound scientific approach to setting dose schedules of antimicrobial drugs is to link PK parameters and variables with appropriate indices of potency, applying the general equation for systemically acting drugs (Fig. 1) (Aliabadi & Lees, 2001, 2002; Toutain & Bousquet‐Melou, 2004; Toutain & Lees, 2004).

The internationally accepted EUCAST) and CLSI methods and standards for *MIC* determinations are based on the use of artificial broths. These are nonbiological growth media, such as Mueller Hinton Broth (MHB), formulated on a pathogen‐by‐pathogen basis. Whilst such media are specifically designed to provide optimal *in vitro* growth conditions, they differ in composition from body fluids. Clinical treatment of disease depends on drug concentration in the biological fluid of the biophase. Concentration in the latter is driven by the plasma concentration of free drug. Therefore, for some drug classes, including tetracyclines and triamilides, it has been proposed that the use of serum to determine *MIC* might be more relevant than reliance on determinations in broths (Nightingale & Murakawa, 2002; Brentnall *et al*., 2013; Honeyman *et al*., 2015; Lees *et al*., 2016; Toutain *et al*., 2017). This is justified by the fact that serum, whilst not identical to biophase fluids, such as pulmonary epithelial lining fluid, is for a given species much closer in composition than artificial broths.

For the same reason, namely closer approximation to bacterial growth conditions *in vivo*, comparative time–kill studies conducted in our laboratory, based on multiples of *MIC*, have been conducted in both broths and serum of the species of interest to establish matrix differences, if any (Illambas *et al*., 2013a,b; Potter *et al*., 2013; Sidhu *et al*., 2014; Lees *et al*., 2015a,b). *In vitro* time–kill data allow classification of killing action as time‐, concentration‐ or co‐dependent and, by modelling the data, PK/PD breakpoints have been generated for a given drug against a given pathogen. In conjunction with PK data and *MIC* distributions of wild‐type organisms, PK/PD breakpoints have been used to predict dosages for a range of target attainment rates (TAR) (Toutain & Lees, 2004; Sidhu *et al*., 2010, 2014; Toutain *et al*., 2017).

The aims of this study were (i) to determine the plasma concentration–time profile for oxytetracycline administered to pigs intramuscularly at the recommended dosage of 20 mg/kg and derive PK variables by noncompartmental analysis; (ii) to integrate *in vivo* PK variables with *in vitro* PD indices of potency (*MIC* and MPC) to determine values of *C*_max_/*MIC*,* C*_max_/MPC, *T* > *MIC*,* T* > MPC and ratios of average concentration (*C*_av_)/*MIC* and *C*_av_/MPC for *A. pleuropneumoniae* and *Pasteurella multocida*; (iii) to model data from time–kill studies of *A. pleuropneumoniae* and *P. multocida* in order to generate PK/PD breakpoint values of *AUC*_24 h_/*MIC* for three levels of bacterial kill, bacteriostasis, bactericidal and 4 log_10_ reduction in inoculum count; (iv) to use PK and PK/PD breakpoints, with serum protein binding data and literature *MIC* distributions in Monte Carlo simulations to estimate dose schedules required for: (i) bacteriostatic and bactericidal levels of kill; (ii) for 50% and 90% TAR; and (iii) for single dosing and daily dosing at steady‐state.

## Materials and Methods

### Origin, storage and selection of bacterial isolates

Twenty isolates of *P. multocida* were supplied by Don Whitley Scientific (Shipley, West Yorkshire, UK). They also supplied three ATCC reference strains, *A. pleuropneumoniae* ATCC 27090, *Enterococcus faecalis* ATCC 29212 and *Escherichia coli* ATCC 25922, to validate *MIC* determinations. Eight isolates of *A. pleuropneumoniae* were supplied by A. Rycroft (Royal Veterinary College, Hawkshead Campus, Hatfield, Herts., UK). All *P. multocida* and *A. pleuropneumoniae* isolates were derived from EU field cases of pig pneumonia. Based on three criteria, six isolates of each species were selected: (i) ability to grow logarithmically in both broth and pig serum; (ii) susceptibility to oxytetracycline in disc diffusion assays (data not shown); and (iii) the highest and lowest broth *MIC*s and four isolates with intermediate *MIC*s, determined using twofold dilutions (data not shown). This initial selection procedure ensured that all isolates could be used in subsequent investigations in both growth media and that they comprised a small but diverse range of susceptible isolates.

### Determination of minimum inhibitory and mutant prevention concentrations

Minimum inhibitory concentrations were determined by microdilution for six isolates each of *A. pleuropneumoniae* and *P. multocida,* except where stated in accordance with CLSI guidelines (2013), using artificial broths, cation‐adjusted Mueller Hinton Broth (CAMHB) for *P. multocida* and Columbia Broth (CB) supplemented with nicotinamide adenine dinucleotide (NAD) for *A. pleuropneumoniae*. CSLI recommended artificial medium for *A. pleuropneumoniae* culture is veterinary fastidious medium; this was replaced for CB as improved bacterial growth was determined and the lack of blood clot formation made *MIC* endpoints easier to establish. To improve accuracy of estimates for each isolate, five sets of overlapping twofold serial dilutions of oxytetracycline were prepared in 96‐well plates and each determination was made in triplicate, instead of the CLSI standard which uses single twofold dilutions. This was considered necessary, because of the small number of isolates of each species used in this study. In addition, the guidelines were adapted using pig serum in place of broth to enable comparison of potency in the two matrices, again with five overlapping sets of twofold dilutions and determinations in triplicate. Methods were as described by Dorey *et al*. (2016).

Mutant prevention concentrations were determined by applying a high count bacterial suspension (1–2 × 10^11^ colony‐forming units (CFU)/mL) onto an agar plate containing drug concentrations 1, 2, 4, 8, 16, 32, 64 and 128 multiples of the *MIC* for each isolate. The concentration ranges were narrowed down two further times. Plates were incubated at 37 °C for 72 h and checked for growth every 24 h. MPC was the lowest oxytetracycline concentration inhibiting bacterial growth completely after 72‐h incubation. The method was validated against that developed by Blondeau *et al*. (2004) and described by Dorey *et al*. (2016). Each experiment was repeated in triplicate for six isolates of each test organism in broth and serum matrices.

### Oxytetracycline pharmacokinetics

Individual animal plasma concentration–time profiles for oxytetracycline were supplied by Norbrook Laboratories Ltd. for two oxytetracycline containing products (Alamycin LA, Norbrook Laboratories, Northern Ireland and Terramycin LA, Pfizer, UK). The study was approved by the company's Ethics Committee and was Good Laboratory Practice compliant. All pigs were Landrace Cross males, aged 2 months. Each product was administered intramuscularly at a dosage of 20 mg/kg. As the two products had been shown to be bioequivalent, the data were pooled, to provide a data set of 14 animals (*n* = 6 for Terramycin LA and *n* = 8 for Alamycin LA) for noncompartmental analysis (NCA) using WinNonLin V6.5 (Pharsight Corporation, Mountain View, CA, USA). Variables calculated were *C*_max_, *C*_av_, *AUC*, area under first moment curve (*AUMC*), time of maximum concentration (*T*_max_), clearance scaled by bioavailability (Cl/F), terminal half‐life (*T*_1/2_) and mean residence time from the time of dosing to the time of last measureable concentration (*MRT*_last_). Three time periods were investigated (0–48 h, 0–24 h and 24–48 h) for determination of average concentrations.

### PK/PD integration

Pharmacokinetic–pharmacodynamic indices, calculated using individual animal PK values and mean *MIC* and MPC values, were as follows: (i) *C*_max_/*MIC*,* T* > *MIC* and *C*_av_/*MIC* ratios for three time periods, 0–24 h, 24–48 h and 0–48 h; (ii) *C*_max_/MPC, *T* > MPC and *C*_av_/MPC ratios for three time periods, 0–24 h, 24–48 h and 0–48 h. *T* > *MIC* and *T* > MPC were calculated using the equation *C* = A*exp(−b*t), where C is the predicted drug concentration at time *t*,* A* is the concentration at *T*_last_, *b* is lambda_z, slope of the terminal phase, calculated by NCA with at least three concentration–time points in the terminal phase. Compared with the prediction using compartmental modelling, the above method generated better fitting, with significantly smaller residuals.

### PK/PD breakpoint determination

**Figure 2:**
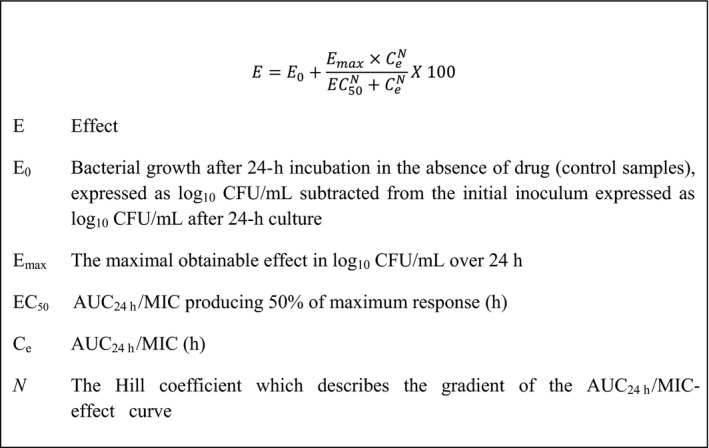
The sigmoidal E_max_ equation used to model time–kill data by nonlinear regression (Lees *et al*., 2015a).

**Figure 3:**
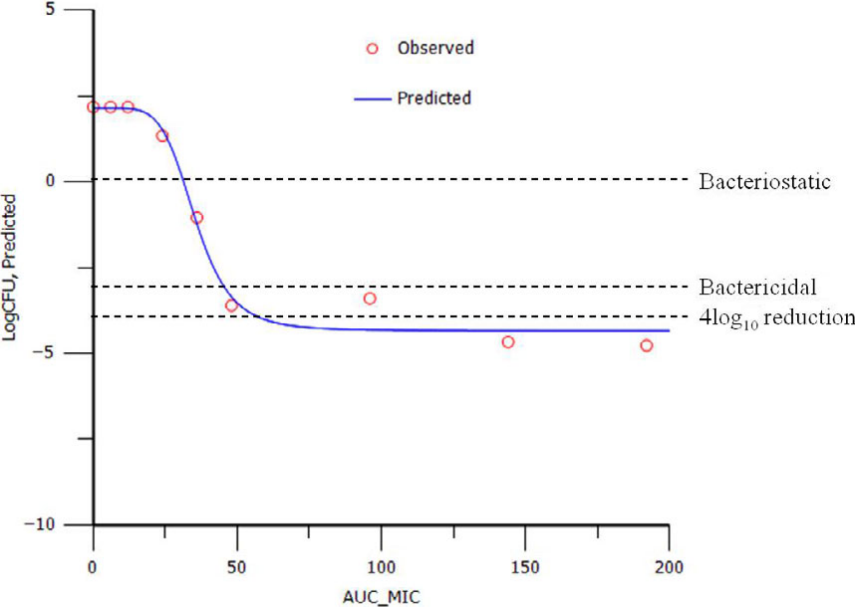
Typical example plot of *AUC*_24 h_/*MIC* vs. change in bacterial count (log_10_ CFU/mL) obtained from *in vitro* time–kill data for oxytetracycline. Each point represents an experimental value showing time–kill 24‐h data point of one isolate, one repeat and one matrix. The curve is the line of best fit based on the sigmoidal E_max_ equation. [Colour figure can be viewed at wileyonlinelibrary.com].

For each pathogen, *in vitro* growth inhibition curves for oxytetracycline were determined using eight multiples of *MIC*, as previously described (Dorey *et al*., 2016). Each test was repeated in triplicate for six isolates each of *A. pleuropneumoniae* and *P. multocida* in both broth (CB and CAMHB, respectively) and pig serum. The lower limit of quantification (LLOQ) was 33 CFU/mL. The sigmoidal E_max_ equation (Fig. 2) was then used to model *AUC*_24 h_/*MIC* data. Using the calculated parameters with this equation, log_10_ change in CFU/mL against *AUC*_24 h_/*MIC* was simulated (Fig. 3). *AUC*_24 h_/*MIC* PK/PD breakpoints were determined for three levels of growth inhibition: *E* = 0, bacteriostatic, that is 0 log_10_ reduction in CFU/mL after 24‐h incubation; *E* = −3, bactericidal, 3 log_10_ reduction in CFU/mL; and *E* = −4, 4 log_10_ reduction in bacterial count.

### Dose determination

For dose prediction using the deterministic approach, average values of PK and PK/PD breakpoint values were used, together with *MIC*_90_ values for *P. multocida* and *A. pleuropneumoniae* for tetracycline obtained from the literature (de Jong *et al*., 2014). *MIC* distribution data for oxytetracycline were not available from a European source (*vide infra*). However, *MIC* and breakpoint interpretation for tetracycline also apply to oxytetracycline, according to CLSI (CLSI, 2013).

**Figure 4:**
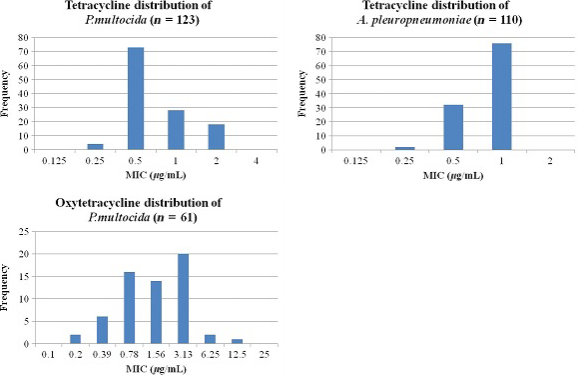
Tetracycline *MIC* frequency distributions for *P. multocida* (*n* = 105) and *A. pleuropneumoniae* (*n* = 110). Data obtained using CLSI methods (de Jong *et al*., 2014). Sampling period covered 2002–2006 from European countries. Oxytetracycline *MIC* frequency distribution for *P. multocida* (*n* = 61). *MIC* data obtained using agar dilution method. Sampling period covered 1994–1998 from Japan. [Colour figure can be viewed at wileyonlinelibrary.com].

**Figure 5:**
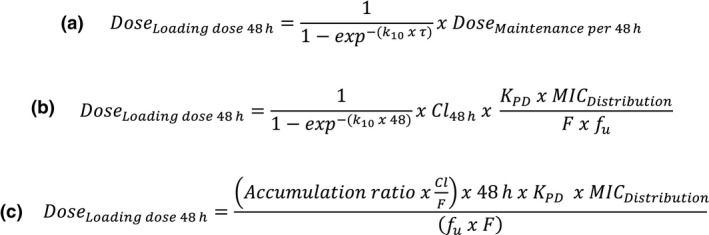
Formulae for calculation of the loading dose for 48‐h duration of action, where equation A can be expressed as equation B and simplified as equation C. *K*_10_ = elimination rate constant; *τ *= dosing interval in h; Cl_48 _= body clearance over 48 h; *K*_PD_ breakpoint = *AUC* divided by 24; *MIC*_Distribution_ = *MIC*s determined from epidemiological surveys; F = bioavailability (from 0 to 1); *f*_u_ = fraction of drug not bound to protein binding.

For dose determination using Monte Carlo simulations, data input comprised: (i) whole body clearance scaled by bioavailability; (ii) free drug concentration in plasma; (iii) *AUC*_24 h_/*MIC* breakpoints derived from time–kill curves by PK/PD modelling; and (iv) *MIC* field distribution data for tetracycline as indicated above (Fig. 4). In addition, for comparative purposes, more limited Japanese oxytetracycline *MIC* distribution data for *P. multocida* in pigs were used (Yoshimura *et al*., 2001). Monte Carlo simulations were based on numerical values and incidence of each input variable and predicted: the daily dose at steady‐state (Fig 5); single loading doses for three time periods, 0–24, 0–48 and 0–72 h; and doses for three levels of bacterial kill, as described by Lees *et al*. (2015a). All dosages were computed using Monte Carlo simulations in Oracle Crystal Ball (Oracle Corporation, Redwood Shores, CA, USA) for TAR of 50% and 90%. For the literature *MIC* distributions, values were corrected using the serum:broth *MIC* ratio, as the reported *MIC* literature values were determined in broth. The probabilities of distribution for the dosage estimation were run for 50 000 simulated trials.

## Results

### Minimum inhibitory and mutant prevention concentrations

Minimum inhibitory concentrations and MPCs were determined for six isolates each of *P. multocida* and *A. pleuropneumoniae,* and each was determined in broth and pig serum. Geometric mean *MIC* values in *μ*g/mL (SD) for *P. multocida* were 0.30 (0.12) and 6.48 (1.45), respectively. Corresponding values for *A. pleuropneumoniae* were 2.50 (0.70) and 2.08 (0.43) (Dorey *et al*., 2016). Mean MPC values in *μ*g/mL (SD) for *P. multocida* were 6.84 (2.76) and 142.8 (33.3) determined in broth and serum, respectively. For *A. pleuropneumoniae*, MPC values were 44.4 (12.63) and 35.1 (6.66) *μ*g/mL, respectively (Dorey *et al*., 2016). For each pathogen, MPC:*MIC* ratios were similar in serum and broth.

### Oxytetracycline pharmacokinetics

From plasma concentration–time data, PK variables were calculated for 14 pigs, comprising data for six animals receiving product A (Terramycin LA) and eight pigs administered product B (Alamycin LA). Each product was administered intramuscularly at a dosage of 20 mg/kg, and the two products were bioequivalent. Table 1 indicates mean values for each variable, determined by noncompartmental analysis.

**Table 1:** Pharmacokinetic variables (mean, standard deviation, *n* = 14) for oxytetracycline

Variable	Units	Mean	SD
*C*_max_	mg/L	5.67	2.40
*AUC*	h·mg/L	84.5	14.7
*AUMC*	h·h·mg/L	1244	221
*T*_max_	h	0.92	1.05
Cl/F	L/h/kg	0.22	0.04
*T*_1/2_	h	12.9	1.83
*MRT*_last_	h	14.7	1.27

Pharmacokinetic variables were determined by noncompartmental analysis for oxytetracycline administered intramuscularly at a dosage of 20 mg/kg. Values are geometric means except for *T*_max_ (arithmetic mean) and *T*_1/2_ (harmonic mean). *C*_max_, maximum concentration; *AUC*, area under plasma concentration–time curve; *AUMC*, area under first moment curve; *T*_max_, time taken to reach maximum concentration; Cl/F, drug clearance scaled by bioavailability; *T*_1/2_, terminal half‐life; *MRT*_last_, mean residence time from the time of dosing to the time of last measureable concentration.

### PK/PD integration

Pharmacokinetic–pharmacodynamic integration established the parameters *C*_max_/*MIC*,* T* > *MIC* and concentration averages (*C*_av_)/*MIC*, over three time periods (0–48, 0–24 and 24–48 h) (Table 2). Integrated values were numerically much greater for broth *MIC* than for serum *MIC* for *P. multocida*;* C*_av0–24_/*MIC* was 9.01 in broth and 0.42 in serum, but for *A. pleuropneumoniae* data were similar between the two media, *C*_av0–24_/*MIC* being 1.08 and 1.30, respectively. For *P. multocida,* the plasma concentration remained above the broth *MIC* for 52.5 h, and *T* > *MIC* for serum was 3.62 h. Corresponding values for *A. pleuropneumoniae* were 11.0 and 15.5 h.

**Table 2:** Integration of pharmacokinetic and pharmacodynamic variables for oxytetracycline for broth and serum *MIC*s (mean and standard deviation)

Organism	Parameter	Units	Broth		Serum	
			Mean	SD	Mean	SD
*Pasteurella multocida*	*C*_av0–48_/*MIC*		5.87	1.02	0.27	0.05
	*C*_av0–24_/*MIC*		9.01	1.75	0.42	0.08
	*C*_av24–48_/*MIC*		3.28	0.81	0.15	0.04
	*C*_max_/*MIC*		18.9	7.99	0.88	0.37
	*T* > *MIC*	h	52.5	5.70	3.62	7.13
*Actinobacillus pleuropneumoniae*	*C*_av0–48_/*MIC*		0.70	0.12	0.85	0.15
	*C*_av0–24_/*MIC*		1.08	0.21	1.30	0.25
	*C*_av24–48_/*MIC*		0.39	0.10	0.47	0.12
	*C*_max_/*MIC*		2.27	0.96	2.73	1.15
	*T* > *MIC*	h	11.0	3.71	15.5	3.38

Individual animal pharmacokinetic data for 14 animals divided by mean *MIC*s for six isolates of each species measured in broth and serum. *C*_av_ = average concentration calculated for time periods 0–48, 0–24 and 24–48 h. *C*_max _= maximum plasma concentration (*μ*g/mL); *T* > *MIC *= time for which plasma concentration exceeds *MIC* (h).

Integration of PK and PD data for MPC is presented in Table 3. All ratios were much lower than the PK:*MIC* ratios, as a consequence of the MPC:*MIC* ratios, which were 22.8:1 (serum) and 22.0:1 (broth) for *P. multocida* and 16.9:1 (serum) and 17.9:1 (broth) for *A. pleuropneumoniae*. *C*_max_/MPC ratios were less than 1 for both pathogens and both matrices.

**Table 3:** Integration of pharmacokinetic and pharmacodynamic variables for oxytetracycline for broth and serum MPCs (mean and standard deviation)

Organism	Parameter	Units	Broth		Serum	
			Mean	SD	Mean	SD
*Pasteurella multocida*	*C*_av0–48_/MPC		0.26	0.04	0.01	0.00
	*C*_av0–24_/MPC		0.40	0.08	0.02	0.00
	*C*_av24–48_/MPC		0.14	0.04	0.01	0.00
	*C*_max_/MPC		0.83	0.35	0.04	0.02
	*T* > MPC	h	0		0	
*Actinobacillus pleuropneumoniae*	*C*_av0–48_/MPC		0.04	0.01	0.05	0.01
	*C*_av0–24_/MPC		0.06	0.01	0.08	0.01
	*C*_av24–48_/MPC		0.02	0.01	0.03	0.01
	*C*_max_/MPC		0.13	0.05	0.16	0.07
	*T* > MPC	h	0		0	

Individual animal pharmacokinetic data for 14 animals divided by mean MPCs for six isolates of each species measured in broth and serum. *C*_av_ = average concentration calculated for time periods 0–48, 0–24 and 24–48 h. *C*_max _= maximum plasma concentration (*μ*g/mL); *T* > MPC = time for which plasma concentration exceeds MPC (h).

### PK/PD modelling

Eight oxytetracycline concentration multiples of *MIC*, ranging from 0.25 to 8 × *MIC*, were used in time–kill studies for six isolates each of *A. pleuropneumoniae* and *P. multocida* for 24‐h incubation periods. This range established bacteriostatic, bactericidal and 4 log_10_ reductions in count at 24 h. Breakpoint values of *AUC*_24 h_/*MIC* producing these levels of growth inhibition are presented in Tables 4 (*P. multocida*) and 5 (*A. pleuropneumoniae*).

**Table 4:** Pharmacokinetic–pharmacodynamic modelling for *Pasteurella multocida* from time–kill data (mean and standard deviation, *n* = 6)

Parameter (units)	Broth		Serum	
	Mean	SD	Mean	SD
Log *E*_0_ (CFU/mL)	2.72	0.30	2.28	0.50
Log E_max_ (CFU/mL)	−4.52	1.35	−5.71	1.62
Log E_max_ − Log *E*_0_ (CFU/mL)	−7.24	1.05	−7.98	1.11
Gamma	4.24	3.05	2.63	1.40
*AUC*_24 h_/*MIC* for bacteriostatic action (h)	26.4	7.88	24.3	6.66
*AUC*_24 h_/*MIC*_50_ (h)	33.4	3.84	39.9	17.3
*AUC*_24 h_/*MIC* for bactericidal action (h)	69.3	42.8	52.3	16.4
*AUC*_24 h_/*MIC* for 4 log_10_ reduction (h)	103.3	54.8	72.4	26.3

E0 = difference in number of organisms (CFU/mL) in control sample in absence of drug between time 0 and 24 h; E_max_ = difference in number of organisms (CFU/mL) in the presence of oxytetracycline between time 0 and 24 h; *AUC*_24_/*MIC*_50 _= concentration reducing count to 50% of the E_max_; Gamma = slope of the curve; detection limit = 33 CFU/mL.

**Table 5:** Pharmacokinetic–pharmacodynamic modelling for *Actinobacillus pleuropneumoniae* from time–kill data (mean and standard deviation, *n* = 6)

Parameter (units)	Broth		Serum	
	Mean	SD	Mean	SD
Log E_0_ (CFU/mL)	2.02	0.37	2.46	0.85
Log E_max_ (CFU/mL)	−6.48	0.37	−5.09	2.38
Log E_max_ − Log E_0_ (CFU/mL)	−8.50	0.00	−7.45	1.53
Gamma	3.89	0.63	3.66	3.02
*AUC*_24 h_/*MIC* for bacteriostatic action (h)	26.3	2.07	33.1	16.7
*AUC*_24 h_/*MIC*_50_(h)	35.6	2.74	40.1	20.4
*AUC*_24 h_/*MIC* for bactericidal action (h)	39.5	3.07	55.4	19.2
*AUC*_24 h_/*MIC* for 4 log_10_ reduction (h)	45.4	4.24	79.7	23.6

E0 = difference in number of organisms (CFU/mL) in control sample in absence of drug between time 0 and 24 h; E_max_ = difference in number of organisms (CFU/mL) in the presence of oxytetracycline between time 0 and 24 h; *AUC*_24_/*MIC*_50 _= concentration reducing count to 50% of the E_max_; Gamma = slope of the curve; detection limit = 33 CFU/mL.

Dividing the *AUC*_24 h_/*MIC* ratios by 24 yields the concentrations, as *MIC* multiples, producing bacteriostatic, bactericidal and 4 log_10_ reductions in count. These are breakpoint *K*_PD_ values; they are more readily comprehended than the term *AUC*_24 h_/*MIC*. *K*_PD_s were 1.10, 2.89 and 4.30, respectively, for *P. multocida* in broth and 1.01, 2.18 and 3.02 for this pathogen in serum. Corresponding values for *A. pleuropneumoniae* were 1.10, 1.64 and 1.89 (broth) and 1.38, 2.31 and 3.32 (serum).

### Average dose determination at steady‐state

Based on mean Cl/F and published values for *MIC*_90_ for tetracycline (de Jong *et al*., 2014), the calculated daily doses for bactericidal level of activity were 1748 mg/kg for *P. multocida* and 535 mg/kg for *A. pleuropneumoniae* (Table 6). However, when the worst‐case scenario was predicted, using the animal with highest clearance (Cl/F = 0.30 L/h/kg) and hence the most rapid clearance, the predicted dose for a bactericidal level of kill was 2381 mg/kg for *P. multocida* and 1269 mg/kg for *A. pleuropneumoniae*.

**Table 6:** Predicted daily doses calculated by deterministic approach

	Daily doses (mg/kg)	
	*Pasteurella multocida*	*Actinobacillus pleuropneumoniae*
Bacteriostatic	813	268
Bactericidal	1748	535
4 log_10_ count reduction	2421	777

*MIC*_90_ for *P. multocida* was 2 *μ*g/mL and 16 *μ*g/mL for *A. pleuropneumoniae* based on tetracycline distribution data (de Jong *et al*., 2014).

### Dose determination by Monte Carlo simulation

Monte Carlo simulations were conducted using the distribution of Cl/F, the distribution of wild‐type *MIC*s for tetracycline (de Jong *et al*., 2014), serum free drug concentration, previously shown to be 29% of total concentration in the pig (Dorey *et al*., 2016), and PK/PD *AUC*_24 h_/*MIC* breakpoint values generated in this study.

Predicted once‐daily doses at steady‐state PKs are presented in Table 7. For *A. pleuropneumoniae* for 50% TAR and a bacteriostatic action, doses were similar, when calculated using broth and serum *MIC*s, 18 and 17 mg/kg, respectively. For a bactericidal level of kill and 90% TAR, corresponding doses were 34 and 43 mg/kg. In marked contrast, for *P. multocida*, there were large differences in predicted doses, depending on whether broth or serum *MIC*s were used. For example, for 50% TAR and a bacteriostatic action, doses were 12 mg/kg (broth) and 232 mg/kg (serum) whilst for 90% TAR and a bactericidal level of kill, corresponding doses were 95 and 1123 mg/kg.

**Table 7:** Predicted daily doses at steady‐state based on tetracycline *MIC* distribution data (de Jong *et al*., 2014) and using broth and serum pharmacodynamic data

	Daily doses (mg/kg)	Target attainment rate			
		50%		90%	
		Broth	Serum	Broth	Serum
*Pasteurella multocida*	Bacteriostatic	12	232	36	730
	Bactericidal	30	357	95	1123
	4 log_10_ reduction	45	421	142	1323
*Actinobacillus pleuropneumoniae*	Bacteriostatic	18	17	23	22
	Bactericidal	26	33	34	43
	4 log_10_ reduction	30	44	40	58

Monte Carlo simulations predicting 50% and 90% target attainment rate dosages at steady‐state for three levels of bacterial kill.

*Pasteurella multocida MIC* distributions for a smaller number of oxytetracycline isolates of Japanese origin (Yoshimura *et al*., 2001) differed from the tetracycline *MIC* distributions of European origin obtained by de Jong *et al*. (2014); for example, the *MIC*_90_s were 12.5 and 2 *μ*g/mL, respectively. Figure 4 demonstrates the *MIC* distribution data after applying epidemiological cut‐off values (ECOFF) to ensure normally distributed data. Therefore, predicted TAR dosages were even higher for the Japanese *MIC* oxytetracycline distributions. Thus, for 50% TAR and bacteriostasis doses were 30 mg/kg (broth *MIC*) and 595 mg/kg (serum *MIC*), whilst for 90% TAR and a bactericidal level of kill, corresponding doses were 189 and 2237 mg/kg (Table 8).

**Table 8:** Predicted daily doses at steady‐state based on oxytetracycline *MIC* distribution data (Yoshimura *et al*., 2001) and using broth and serum pharmacodynamic data for *Pasteurella multocida*

		Predicted daily doses (mg/kg)	Target attainment rate
		50%	90%
Broth	Bacteriostatic	30	72
	Bactericidal	78	189
	4 log_10_ reduction	116	282
Serum	Bacteriostatic	595	1453
	Bactericidal	916	2237
	4 log_10_ reduction	1079	2635

Monte Carlo simulations predicting 50% and 90% target attainment rate dosages at steady‐state for three levels of bacterial kill.

The single oxytetracycline doses predicted to achieve three levels of kill (0, 3 and 4 log_10_ decreases in count) for three durations of action (24, 48 and 72 h) are presented in Table 9. For a duration of action of 24 h and a bacteriostatic level of kill, the 50% TAR single dosages, using serum/broth oxytetracycline *MIC*s and tetracycline wild‐type distribution data, were 333/17 mg/kg (*P. multocida*) and 21/25 mg/kg (*A. pleuropneumoniae*). Bactericidal levels of kill for a duration of action of 48 h at 90% TAR, also using tetracycline *MIC* distributions and serum/broth *MIC* data, were 3462/209 mg/kg for *P. multocida* and 84/76 mg/kg for *A. pleuropneumoniae*. Doses were higher for *P. multocida* when predicted using oxytetracycline distribution data (Table 10).

**Table 9:** Single doses for 24‐, 48‐ and 72‐h durations of action based on tetracycline *MIC* distribution data (de Jong *et al*., 2014) and using broth and serum pharmacodynamic data

	Dose duration	Level of bacterial kill	Target attainment rate			
			Broth		Serum	
			50%	90%	50%	90%
*Pasteurella multocida*	0–24 h	Bacteriostatic	17	50	333	1012
		Bactericidal	43	131	716	2175
		4 log_10_ reduction	65	196	992	3014
	0–48 h	Bacteriostatic	25	80	512	1611
		Bactericidal	67	209	1102	3462
		4 log_10_ reduction	99	312	1526	4796
	0–72 h	Bacteriostatic	36	112	719	2270
		Bactericidal	93	295	1545	4879
		4 log_10_ reduction	139	439	2141	6759
*Actinobacillus pleuropneumoniae*	0–24 h	Bacteriostatic	25	33	21	28
		Bactericidal	37	49	41	55
		4 log_10_ reduction	43	57	60	80
	0–48 h	Bacteriostatic	39	51	33	42
		Bactericidal	58	76	64	84
		4 log_10_ reduction	67	87	93	122
	0–72 h	Bacteriostatic	54	71	45	59
		Bactericidal	81	106	90	118
		4 log_10_ reduction	94	122	131	171

Monte Carlo simulations predicting 50% and 90% target attainment rates for three levels of bacterial kill and three action durations.

**Table 10:** Single doses for 24‐, 48‐ and 72‐h durations of action based on oxytetracycline *MIC* distribution data (Yoshimura *et al*., 2001) and using broth and serum pharmacodynamic data

Dose duration	Level of bacterial kill	Target attainment rate			
		Broth		Serum	
		50%	90%	50%	90%
0–24 h	Bacteriostatic	42	103	841	2088
	Bactericidal	109	28	1807	4487
	4 log_10_ reduction	163	404	2504	6215
0–48 h	Bacteriostatic	65	159	1305	3211
	Bactericidal	169	417	2804	6899
	4 log_10_ reduction	417	621	3885	9557
0–72 h	Bacteriostatic	91	223	1838	4503
	Bactericidal	238	584	3949	9676
	4 log_10_ reduction	355	871	5471	13 403

Monte Carlo simulations predicting 50% and 90% target attainment rates for three levels of bacterial kill and three action durations.

## Discussion

The objective of this study was to predict doses for oxytetracycline for the pig pneumonia pathogens, *P. multocida* and *A. pleuropneumoniae* based on principles of PK/PD integration and modelling and the application of Monte Carlo simulation.

### Pharmacokinetics

Plasma concentration–time data for oxytetracycline were determined for a small number (14) of healthy pigs of the same age and breed and for two products. In extending the present findings, it will, in future studies, be appropriate to obtain data from greater animal numbers of both sexes and differing ages and breeds and in diseased as well as healthy pigs. Thus, population PK data, derived from field studies, can be used to determine the impact of disease on clearance and bioavailability, Cl/F being a major determinant of dosage predictions (Toutain *et al*., 2017). Nevertheless, the more limited and less variable data used in this study illustrate the principles of integrating PK with PD data to predict TAR dosages.

### Pharmacodynamics

In this study, a small number of isolates, six for each of two pathogens, were used in PK/PD integration and modelling approaches to dose determination; the question arises whether they are representative of the much larger number of wild‐type isolates of these organisms. Clearly, whilst sample size was small, it nevertheless may be noted that: (i) the unavoidable inaccuracy of each individual *MIC* potency estimate was reduced from potentially approaching 100% (as when single two‐dilutions are used using CLSI standards) to not greater than 20% by the use of five overlapping sets of twofold dilutions; (ii) mean broth *MIC*s for oxytetracycline in this study was 2.5 *μ*g/mL (*A. pleuropneumoniae*), and this may be compared with broth *MIC*_50_ and *MIC*_90_ values of 1.0 and 16.0 *μ*g/mL (*A. pleuropneumoniae*) reported for tetracycline by de Jong *et al*. (2014). Corresponding values for *P. multocida* were 0.30 *μ*g/mL (this study) and 0.50 and 2.0 *μ*g/mL for tetracycline (de Jong *et al*., 2014); and (iii) the present data were supported by a second potency index, namely MPC, and the proportional increase in potency (MPC:*MIC* ratio) was similar for determinations in both broth and serum for both organisms.

### PK/PD integration

Pharmacokinetic–pharmacodynamic integration provided an initial and tentative approach for evaluating efficacy of currently recommended oxytetracycline dose schedules for the pathogens, *P. multocida* and *A. pleuropneumoniae*. Thus, plasma oxytetracycline *T* > *MIC* values over the period 0–48 h were 11.0 and 15.5 h in broth and serum, respectively, for *A. pleuropneumoniae*. Corresponding *T* > *MIC*s for *P. multocida* were 52.5 h (broth *MIC*) and 3.6 h (serum *MIC*). Therefore, prediction of efficacy from PK/PD integration was broadly similar for potency measured in serum and broth for the former species, but markedly different for the latter pathogen. If it is accepted that potency measured in serum is more likely to reflect activity in the biophase (pulmonary epithelial lining fluid for these pathogens) then some efficacy in clinical subjects against *A. pleuropneumoniae* (especially if pathogen load in the biophase is low to moderate) might be expected. For *P. multocida*, on the other hand, the expectation would be of very limited or no efficacy, insofar as it depends solely on a direct killing action of oxytetracycline. However, in the absence of high‐quality clinical trial data, the integrated PK/PD index (and its numerical value) that best correlates with bacteriological cure is not known and can only be surmised.

As MPCs were 17‐ to 23‐fold greater than corresponding *MIC*s and as MPC:*MIC* ratios were, moreover, independent of growth matrix, the integration of PK with PD data indicated that plasma concentrations of oxytetracycline could not be achieved, with clinical dosages of oxytetracycline in healthy pigs, which would ensure the eradication of the least sensitive subpopulation in a given colony for both bacterial species.

### PK/PD modelling and breakpoint determination

Pharmacokinetic–pharmacodynamic modelling is an advance on PK/PD integration, in that it provides breakpoint values for a given drug against a particular pathogen obtained from a given animal species. In addition, it describes the whole sweep of the concentration–effect relationship, so that any predetermined level of *in vitro* activity, ranging from bacteriostasis to virtual eradication (indicated by the breakpoint *AUC*_24 h_/*MIC* index) can be determined. In this study, breakpoint values for each level of growth inhibition, 0 log_10_, 3 log_10_ and 4 log_10_ reductions in count, were similar between the two growth matrices; this is not unexpected as, although *MIC*s in broth and serum differed for *P. multocida*, the breakpoint values are based on *MIC* multiples. However, there were trends for increased interisolate variability with increasing level of kill, especially for *P. multocida* (Tables 4 and 5). This variability might translate to clinical efficacy variability for this pathogen, although the dose determination data imply little or no efficacy from a direct killing action of oxytetracycline (*vide infra*).

### Dosage prediction

The deterministic approach to dosage prediction provides a useful estimate of once‐daily doses at steady‐state, based on *MIC*_90_ as a single value and the average values for other variables. It does not take into account, however, either of variability or incidence of each input variable, such as clearance or *AUC*/*MIC*, as dose is calculated using mean values. Nevertheless, it comprises an initial evaluation prior to use of Monte Carlo simulations to estimate population doses for each selected TAR, which is a dose encompassing a given percentile of the target population, for example, 50% or 90% and for three levels of bacterial kill. Moreover, TAR once‐daily doses have been determined for maintaining efficacy under steady‐state conditions, as well as for TAR single doses over 24, 48 or 72 h time periods, that is either for a single or a loading dose, in the latter case possibly to be repeated after a selected time interval.

The Monte Carlo simulation with PK/PD modelling basis for predicting dosage, for subsequent evaluation in clinical trials, has the advantage of taking into account all important PK and PD variables impinging on bacteriological kill. Furthermore, basing potency estimates on serum as a growth matrix may have greater relevance to *in vivo* conditions than *MIC* determined in broths, whilst recognizing that serum, although similar, is not identical in composition to the biophase at infection sites. Moreover, Monte Carlo simulations predict doses, allowing for incidence within *MIC* distributions and encompassing best, worst and all intermediate case scenarios for distributions of Cl/F and breakpoint *AUC*_24 h_/*MIC* ratios.

These clear advantages have led to the widespread application of Monte Carlo simulations to predict dosages (Nielsen *et al*., 2011; Martinez *et al*., 2012; Mouton *et al*., 2012; Brentnall *et al*., 2013; Nielsen & Friberg, 2013; Papich, 2014; Rey *et al*., 2014; Sidhu *et al*., 2014; Lees *et al*., 2015a; Toutain *et al*., 2017). Nevertheless, there are inevitable limitations to study methodology. For example, for this study *MIC* distribution data were extracted from the literature, and the number of isolates was limited (230 for *P. multocida* and 220 for *A. pleuropneumoniae*). Moreover, they were obtained from one geographical location (Europe) for tetracycline, a drug with likely similar but not necessarily identical antimicrobial profile to oxytetracycline. For CLSI (2013), tetracycline is the class representative but it is proposed that *MIC* tetracycline distribution also applies to oxytetracycline. Comparing tetracycline *MIC* distribution for European isolates against Japanese oxytetracycline *MIC* distribution for *P. multocida* indicated *MIC* ranges of 0.2–12.5 *μ*g/mL for oxytetracycline (including likely resistant isolates) and 0.25–32 *μ*g/mL for tetracycline (including likely resistant isolates). *MIC*_50_ and *MIC*_90_ differed, 1.56 and 12.5 *μ*g/mL, respectively, for oxytetracycline, and corresponding values for tetracycline were 0.5 and 2.0 *μ*g/mL

Whilst the interisolate variability in PK/PD breakpoint values was small to moderate in the present study, estimates were based on only six isolates for each species. The time–kill studies used fixed drug concentrations (eight multiples of *MIC*) for a fixed time period. In clinical use, on the other hand, plasma drug concentrations would first increase and then decrease after intramuscular dosing, exposing organisms to a continuously variable concentration. In future studies, these concerns could be addressed by increasing numbers of isolates in field distribution studies and in PK/PD breakpoint estimation studies. Moreover, exposure of organisms to varying drug concentrations could be addressed by use of *in vitro* pump (e.g. hollow fibre) methods to simulate *in vivo* patterns of change in concentration with time (Cadwell, 2012).

Finally, the methodology in this study also does not incorporate the contribution to pathogen elimination by the body's natural defence mechanisms in immune‐competent clinical subjects, nor does it allow for additional potentially beneficial properties of antimicrobial drugs, such as immunomodulatory and anti‐inflammatory actions (*vide infra*).

The depot formulations of oxytetracycline licensed to treat porcine respiratory diseases recommend a single dose of 20 mg/kg to achieve up to four days action duration. Based on serum *MIC*s, the predicted 90% TAR dosage against *P. multocida* was 1123 mg/kg daily (bactericidal action at steady‐state) and 3462 mg/kg (bactericidal action as single dose and 48‐h duration). In similar studies for *P. multocida* of calf origin, corresponding doses were 921 and 1523 mg/kg (Lees *et al*., 2016). These differences for the two animal species and the same bacterial species are explained by: similar values for the *AUC*_24 h_/*MIC* bactericidal breakpoints; approximately twofold higher values of Cl/F in the pig; differing free drug fractions in serum (29% in the pig, 48% in the calf); and differing *MIC* distribution patterns. For both animal species, however, it is clear that practicable doses to achieve a direct killing action of oxytetracycline are likely not to be attainable.

For *A. pleuropneumoniae*, predicted dosages for 90% TAR and bactericidal level of kill were 42.5 mg/kg (daily dose at steady‐state) and 84.3 mg/kg (single dose with 48‐h duration). Whilst still exceeding the recommended dose regimen, these lower doses for this pathogen compared to *P. multocida* reflect the broth:serum potency differences for the two bacterial species. Serum:broth *MIC* ratios, after correcting for protein binding in serum, for six isolates of each species were 6.30:1 (*P. multocida*) and 0.24:1 (*A. pleuropneumoniae*) (Dorey *et al*., 2016).

In conclusion, it might be noted that tetracyclines have activities other than direct killing actions, and clinically, these might contribute to or even account for efficacy.
